# Barriers to Nutrition Promotion in Private Secondary Schools in Kolkata, India: Perspectives of Parents and Teachers

**DOI:** 10.3390/ijerph15061139

**Published:** 2018-06-01

**Authors:** Neha Rathi, Lynn Riddell, Anthony Worsley

**Affiliations:** Institute for Physical Activity and Nutrition, School of Exercise and Nutrition Sciences, Deakin University, Geelong VIC 3220, Australia; lynn.riddell@deakin.edu.au (L.R.); anthony.worsley@deakin.edu.au (A.W.)

**Keywords:** nutrition promotion, secondary schools, canteens, India, survey

## Abstract

School-based nutrition promotion is critical to the development of healthy eating habits in adolescents. Unfortunately, most Indian secondary schools do not support healthy eating among adolescents. Therefore, this study was designed to understand the perspectives of teachers and parents regarding the potential barriers to nutrition promotion in Indian secondary schools. Thirty-two teachers and 280 parents from five private English-speaking secondary schools in Kolkata, India took part in a cross-sectional survey. The paper-based survey instrument included both quantitative and qualitative items which were derived from previously published evidence. Descriptive and chi-square analyses were conducted on the quantitative data. Qualitative data were evaluated by thematic data analysis underpinned by the template analysis technique. Five main barriers to nutrition promotion emerged from the study. These included the perceived strong preference for energy-dense, nutrient-poor foods among students; lack of government canteen guidelines; limited engagement of parents, teachers, and adolescents in canteen operations; the widespread display of eye-catching food advertisements; and poor knowledge among canteen personnel with regards to healthy food preparation. These findings about the potential barriers to nutrition promotion provide useful directions for healthy school food policy implementation. Successful implementation of healthy school food policies can inculcate lifelong healthy eating habits among adolescents.

## 1. Introduction

Over the last decade, adolescent obesity has emerged as a major public health crisis in India [1,2]. Obesity is associated with a number of chronic degenerative diseases such as cardiovascular diseases, type 2 diabetes, arthritis, impaired mobility, depression, and certain carcinomas [3,4]. The treatment of these life-threatening conditions is expensive; and is a major threat to national development [5]. This has prompted calls for the development of prevention strategies that address adolescent obesity and its associated comorbidities.

Unhealthy eating is an important determinant of this emerging health crisis [4,6]. In fact, most urban Indian adolescents appear to consume nutritionally deficient diets with inadequate intakes of fruits and vegetables and over consumption of energy-dense, nutrient-poor foods and sugar-sweetened beverages [7,8,9]. Since, adolescent dietary patterns are significant determinants of health and nutritional status in adulthood [10], it is important to develop and implement strategies that support healthy eating from an early age.

Globally, schools have been identified as powerful platforms for supporting physical and psychological well-being as well as social and academic development in young people [11,12,13]. However, Indian schools have been criticized for inculcating unhealthy eating habits among pupils [13,14,15]. Indeed, recent studies have shown that there is heavy marketing of energy-dense, nutrient-poor foods (e.g., French fries) in Indian schools which have limited students’ exposure to healthy foods [14,15,16]. Furthermore, many supply foods at inflated prices, handle foods in an unhygienic manner, and produce misleading nutrition information across the student community [14,17]. This negative assessment of Indian school food services reflects the absence of school food policies [14,18,19]. Unfortunately until now, Indian governments both at the central and state levels have not developed any healthy school food policies for the promotion of healthy eating [14]. Even the World Health Organization’s popular school-based program—the ‘Health Promoting School’ program (HPS) [20]—is not widely implemented in India [18,19,21]. This highlights the importance of designing and implementing healthy school food policies for Indian schools to support healthy eating among students.

Healthy school food policies should be an integral part of the school food environment [22,23]. A school food policy is a written document that reinforces positive changes in the school food environment (e.g., the supply of healthy foods, skills-focused nutrition education) to foster healthy eating among students and staff [22]. Substantial international evidence supports the efficacy of school food policies in improving the eating habits of young people at school [23,24,25]. However, Indian schools have failed to adopt healthy food policies [14,18,19] thus inhibiting nutrition promotion in Indian schools. This situation underscores the need to explore the barriers that inhibit nutrition promotion in Indian schools. Therefore, the current investigation aimed to understand the views of Indian parents and teachers about the possible barriers to nutrition promotion. The perspectives of parents and teachers are critical to this investigation as both these groups play an important role in the successful implementation of school-based nutrition programs [26,27].

## 2. Materials and Methods

### 2.1. Research Design and Sampling 

This study was a cross-sectional survey that builds on the lead researcher’s (NR) previous food environment and nutrition education research [13,14,17]. This was primarily focused on qualitative examination of key stakeholders’ views of the current food and nutrition curriculum, and food services and policies and adolescent food consumption in Kolkata private secondary schools. The present study was designed on the basis of this earlier research to understand the various barriers to nutrition promotion in Indian schools.

Convenience sampling was employed to select five private English-speaking schools in Kolkata metropolitan area. Considering, the high prevalence of obesity among private school pupils compared with public school pupils [15,28], only private schools were selected for this study.

Parents of adolescents studying in year 9 and secondary school biology and home science (i.e., Home Economics) educators were invited to participate in the study. Both of these stakeholder groups were recruited because they are generally recognized as key and meaningful players in the school system [26,27]. Moreover, both these groups were likely to be well-informed about various nutrition promotion issues in Indian secondary schools [14,17]. Methodological details for this study have also been described elsewhere [29,30].

### 2.2. Survey Instrument

An anonymous, self-reported, survey instrument (School Food Landscape Questionnaire—SFLQ) was developed to explore the food and nutrition situation in Indian secondary schools. This paper-based tool included both close-ended questions (n = 115) as well as open-ended questions (n = 8) on nutrition education, culinary skill acquisition, school canteen and policies, adolescent eating behavior, and demographics. These measures were derived from recently published qualitative [13,14,17] and quantitative studies [16,31] on Indian schools.

This article focuses on quantitative as well as qualitative measures pertaining to the barriers to nutrition promotion in Indian secondary schools. These barriers were highlighted by adolescents, parents, teachers, and school principals in our qualitative study exploring the school food environment and canteen policies in Indian schools [14]. Therefore, to confirm the generalizability of these findings with a larger sample, this cross-sectional study was designed. Five statements were used to understand stakeholders’ perspectives of the various barriers to nutrition promotion (Table 1). Responses to these statements were recorded on a five-point Likert response scales ranging from “strongly disagree” (coded as 1), “disagree” (2), “neutral” (3), “agree” (4), to “strongly agree” (5). An open-ended question was also presented to the respondents: “*In addition to the above mentioned barriers, are there any other barriers to making improvements in secondary schools? If so, please describe them and suggest how you would overcome them.*”

### 2.3. Procedure

A pilot study was conducted with nine educators and 21 parents to check the comprehensibility, length, and content of the questionnaire. As a result, only minor modifications (e.g., question order; text clarification) were made to the questionnaire. Data generated from the pilot survey were not combined with the main survey data, and the school involved in the pilot survey was excluded from the main survey.

To proceed with the main survey, an in-depth description of the data collection methods was explained to the principals of the five recruited schools. Subsequently, the principals completed and returned the organizational consent form to the lead researcher (NR), indicating their permission for conducting the survey. On the school premises, 35 teachers were provided with the Plain Language Statement and Consent Form, the questionnaire and an envelope. The school authorities attached the recruitment packs for the parents to the diaries of all year nine students (n = 309).

The respondents were asked to return the completed questionnaire and consent form in sealed envelopes to the school authorities within one week. A reminder was sent to them if they did not submit the questionnaire within the stipulated time. Three weeks after the initiation of the survey, the lead researcher (NR) collected all the sealed envelopes from the school authorities. This study was conducted between August and November 2016. The stakeholders did not receive any gifts or inducements for completing the questionnaire. This study was approved by Deakin University’s Health Ethics Advisory Group (HEAG-H 127_2016). Written informed consent was obtained from all respondents.

### 2.4. Data Analysis

The Statistical Package for Social Sciences (SPSS) version 22.0 was used to analyze the quantitative data. Descriptive statistics comprising means, frequencies, and percentages were computed. After inspection of the distribution of the data, the five-point response scales were merged into three-point response scales i.e., ‘Strongly disagree/Disagree’; Neutral; and ‘Strongly Agree/Agree’ during data analysis. Parents’ and teachers’ perceptions were then compared by conducting cross-tabulation analyses.

The NVivo 10 software (QSR International Pvt Ltd., 2010, Melbourne, Australia) along with some manual coding was used to thematically analyse responses to the open-ended question. The thematic data analysis was underpinned by the Template Analysis Technique [32]. This data-driven technique involves repeated reading of respondents’ statements and subsequent extraction of relevant concepts. This informed the development of themes. Quotations were then selected to represent these emergent themes.

## 3. Results

### 3.1. Response Rate

Out of 344 eligible respondents (35 teachers; 309 parents), 312 respondents completed the survey; a response rate of 91%. In addition to the high response rate, this sample provided adequate power (83%) for the study, an effect size of 0.2 (Cohen’s w employed in cross-tabulation analyses), at a significance level of 0.01. It must be noted that there were no missing data as all the questions were answered by the respondents.

### 3.2. Sociodemographic Characteristics of the Sample

The sample was aged between 25 and 55 years with the mean age being 41.9 years (SD = 4.5 years). About, two-thirds (68.6%) of the respondents were women; Hinduism was the most popular reported religious affiliation (80.1%). The majority of the respondents (88.6%) had attended university.

### 3.3. Quantitative Findings

The main reported barriers to nutrition promotion in Indian secondary schools were the perceived liking of unhealthy foods by the adolescents (81.7%), followed by an absence of any government food policy mandate (61.9%), and school authorities’ reluctance to involve other stakeholders in school food services (49.0%; Table 1). A substantial minority (44.6%) agreed there was immense pressure from canteen staff to sell nutrient-poor foods and that pupils would violate any canteen guidelines (39.7%).

The cross-tabulation analyses indicated that significantly higher proportions of parents than teachers agreed with the statement that there was immense pressure from canteen personnel to sell nutrient-poor foods and that the school management was hesitant to engage parents, teachers and students in canteen services (Table 1). No other statistically significant differences in the responses were observed.

### 3.4. Qualitative Findings

The respondents identified three main themes associated with the barriers to nutrition promotion in Indian schools. These themes are discussed below:

- Mass media has a negative effect on adolescent eating habits

Food advertisements were recognized as one of the significant barriers preventing nutrition promotion in schools. The majority of the sample (92.9%) noted that food advertisements were so ubiquitous that they strongly influenced the intake of unhealthy foods in young people (All the percentages detailed in this section denote the percentages of survey respondents reporting specific themes).

*“Adolescents are often victims of foods advertised on TV and other forms of media that they tend to consume those kind of foods……”*

(Teacher 8)

*“One additional barrier could be mass media……”*

(Parent 11)

- *Lack of healthy cooking knowledge among canteen staff*

The lack of appropriate knowledge regarding healthy meal preparation among canteen staff was cited as another potential barrier to nutrition promotion in school settings. Because of their inadequate knowledge, canteen staff were unable to prepare nutritious foods for students.

“You know there is a lack of awareness among the canteen staff regarding the dietary requirements of adolescents. So how can you expect them to prepare healthy foods?”

(Parent 126)

“I feel the catering staff needs the training to prepare nutritious foods for students….”

(Teacher 4)

- *Parents have inadequate nutrition knowledge*

Only a minority of respondents (14.7%), mainly teachers (78% of teachers) raised this theme. They felt that inadequate parental counselling inhibited nutrition promotion in schools. The teachers observed that parents did not promote healthy eating practices at home. For example, one teacher described this inadequacy as follows:

“Parents nowadays are very busy and therefore they mostly take their kids to restaurants for dinner. Unfortunately, kids develop the taste for fast food and only want to have those kinds of food even in school!”

(Teacher 15)

Similarly, one parent also noted:

“We should also follow what we are preaching to our children.”

(Parent 18)

## 4. Discussion

The present investigation reports novel findings about the barriers to nutrition promotion in private Indian secondary schools. There were five main findings: the perceived preference for nutrient-poor foods among secondary school students; lack of a government mandate for school food policies; limited engagement of parents, teachers, and adolescents in canteen operations; the widespread display of eye-catching food advertisements; and poor knowledge among canteen personnel about healthy food preparation.

The majority of the respondents criticized the secondary school students’ liking for energy-dense, nutrient-poor foods. Indeed, empirical evidence from both developed [33,34] and developing economies including India [13,35,36] suggests that the palatability of energy-dense, nutrient-poor foods over nutritious foods is highly appealing and this leads to its excessive consumption among adolescents. Since, such excessive consumption of nutrient-poor foods can result in overweight and obesity [4,6], Indian schools should supply palatable nutritious foods to students, as recommended in the past [37,38].

The lack of a government mandate regarding school food policy was also cited as a potential barrier to school-based nutrition promotion. Previous local studies have also drawn attention to this inadequacy [14,18,19]. The absence of strong government support for school food policies may partly explain the excessive promotion of nutrient-poor foods in Indian school canteens [13,15,16]. There is ample evidence to support the positive influence of healthy school food polices on young people’s eating habits [23,24,25]. India’s lack of government support is not unique. Schools in the UK [39], Malaysia [36], and Sri Lanka [35] do not support the implementation of school food policies. This lack of support is compounded by the violation of food policy guidelines by key stakeholders (e.g., teachers, students) [14]. Involvement of all the key stakeholders in the development and implementation of school food polices may help to resolve this situation.

Consistent with our previous qualitative investigation [14], this study showed that Indian school authorities were less likely to entertain the engagement of parents, teachers, and students in school canteen operations as reported by half of the sample. Such limited involvement of key stakeholders is a significant barrier to implementation of healthy school food policies [23,27]. Unlike Indian schools, studies conducted in schools in Portugal [40], China [41] and other countries have shown that schools encourage the participation of all key stakeholders including parents, teaching staff, and pupils in their canteen operations [23,26,42,43,44]. Considering this strong support in favor of the engagement of parents, teachers, and students in school operations, Indian schools might consider this nutrition promotion strategy in improving the healthiness of their canteen services.

The widespread display of food commercials in electronic, print, and social media was viewed as a potential barrier to nutrition promotion. The content of food advertising worldwide is primarily limited to nutrient-poor foods including, confectionery, salty snacks, and sugar-sweetened beverages [45,46,47,48]. Unfortunately, this has negative health repercussions [49,50,51]. Commercials promoting healthy foods are seldom advertised [46]. This underscores the need for the creation of healthy media environments for young people. Perhaps, Indian schools should only display advertisements associated with nutritious foods on their school notice boards and websites to encourage healthy dietary practices.

Nearly half of the sample reported that school managements were compelled by canteen proprietors to supply energy-dense, nutrient-poor foods, a barrier consistently cited by the school principals in our previous qualitative inquiry [14]. Indian private school canteens are usually outsourced to commercial enterprises resulting in a lack of effective contribution by the school management in menu planning [14]. Similarly, Brazilian [52] and Dutch [53] schools hire commercial catering businesses, and exert minimum influence over canteen operations.

The respondents felt that canteen personnel were mostly ignorant and unaware of healthy eating practices. This limitation might be minimized by offering training in food selection and marketing to canteen staff [52,54,55]. Canteen personnel can play an important role in influencing students’ food choices at the point-of-purchase [55]. For example, Fulkerson and colleagues reported that 50% of the school food service staff (n = 235) from 16 US middle schools believed that influencing pupils’ food choices was a part of their routine job and nearly four-fifths of them were comfortable in guiding the students on what to purchase [55]. In view of these findings, it is important to train Indian canteen personnel in nutrition and food management courses, an initiative successfully implemented by schools in New South Wales, Australia [56] during the ‘Fresh Tastes @ School’ program.

The potential limitations of this research investigation include its cross-sectional study design that prevents causal associations between variables being made. Perhaps, longitudinal or experimental designs could be employed in future to allow for the identification of causal associations. The use of convenience sampling in the selection of schools is another limitation. Logistic limitations prevented the use of random sampling. The selection of private schools in Kolkata could have resulted in regional bias, further limiting the generalizability of the survey findings. To test the generalizability of the present findings, the survey should be replicated in public schools as well as in other urban and rural regions in India. Future research should also focus on examining the views of other key stakeholders including school principals, canteen staff, and government officials to allow for a more comprehensive assessment of the potential barriers to nutrition promotion in Indian secondary schools.

Nevertheless, this cross-sectional survey had several strengths. The findings are novel as it is the first survey to explore teachers’ and parents’ views of the potential barriers to nutrition promotion in Indian secondary schools. An additional strength was the use of qualitative methodology which provided a comprehensive, in-depth analysis of the potential barriers. The high response rate (90.7%) added further strength to the survey.

## 5. Conclusions

The survey respondents identified a number of potential barriers to nutrition promotion in Indian secondary schools. Both the qualitative and quantitative findings highlight the need to involve all the key stakeholders, as well Indian governments at the central and state level, in the development and implementation of healthy school food policies. Provision of effective training for school canteen staff about healthy meal preparation is also required. These and similar initiatives have the potential to positively impact the eating habits of Indian secondary school students.

## Figures and Tables

**Table 1:** Respondents’ views of the barriers to nutrition promotion in Indian secondary schools (% Agree *).

	Parents % (n = 280)	Teachers % (n = 32)	Total % (n = 312)	χ^2 #^	df	*p*-Value
Adolescents like unhealthy foods	81.4 (228)	84.4 (27)	81.7 (255)	0.19	2	0.91
There is immense pressure from the school canteen personnel to sell unhealthy foods	47.5 (133)	18.8 (6)	44.6 (139)	14.31	2	<0.01
There is lack of any Indian government mandate regarding school canteen policy	63.9 (179)	43.8 (14)	61.9 (193)	5.42	2	0.67
The school management is reluctant towards the participation of student, teacher and parent in school canteen operations	52.9 (148)	15.6 (5)	49.0 (153)	16.60	2	<0.01
Students could probably violate the canteen policy rules	40.0 (112)	37.5 (12)	39.7 (124)	0.61	2	0.74

Agree * = agree (4) + strongly agree (5); **χ^2^** ^#^ is used for comparing parents vs. teachers.
